# Supplementary material for: Quality of Automated Stereotactic Radiosurgery Plans in Patients with 4 to 10 Brain Metastases
Source: Cancers (Basel). 2021 Jul 10;13(14):3458. doi: 10.3390/cancers13143458 (PMC8307043; doi:10.3390/cancers13143458)
Supplement: Supplementary file 1 [file cancers-13-03458-s001.zip › cancers-1277954 Supplementary Material.pdf]

Supplementary Materials: Quality of Automated Stereotactic Radiosurgery Plans in Patients with 4 to 10 Brain Metastases

Anna Petoukhova, Roland Snijder, Rudolf Wiggenraad, Linda de Boer-de Wit, Ivonne Mudde-van der Wouden, Mireille Florijn and Jaap Zindler

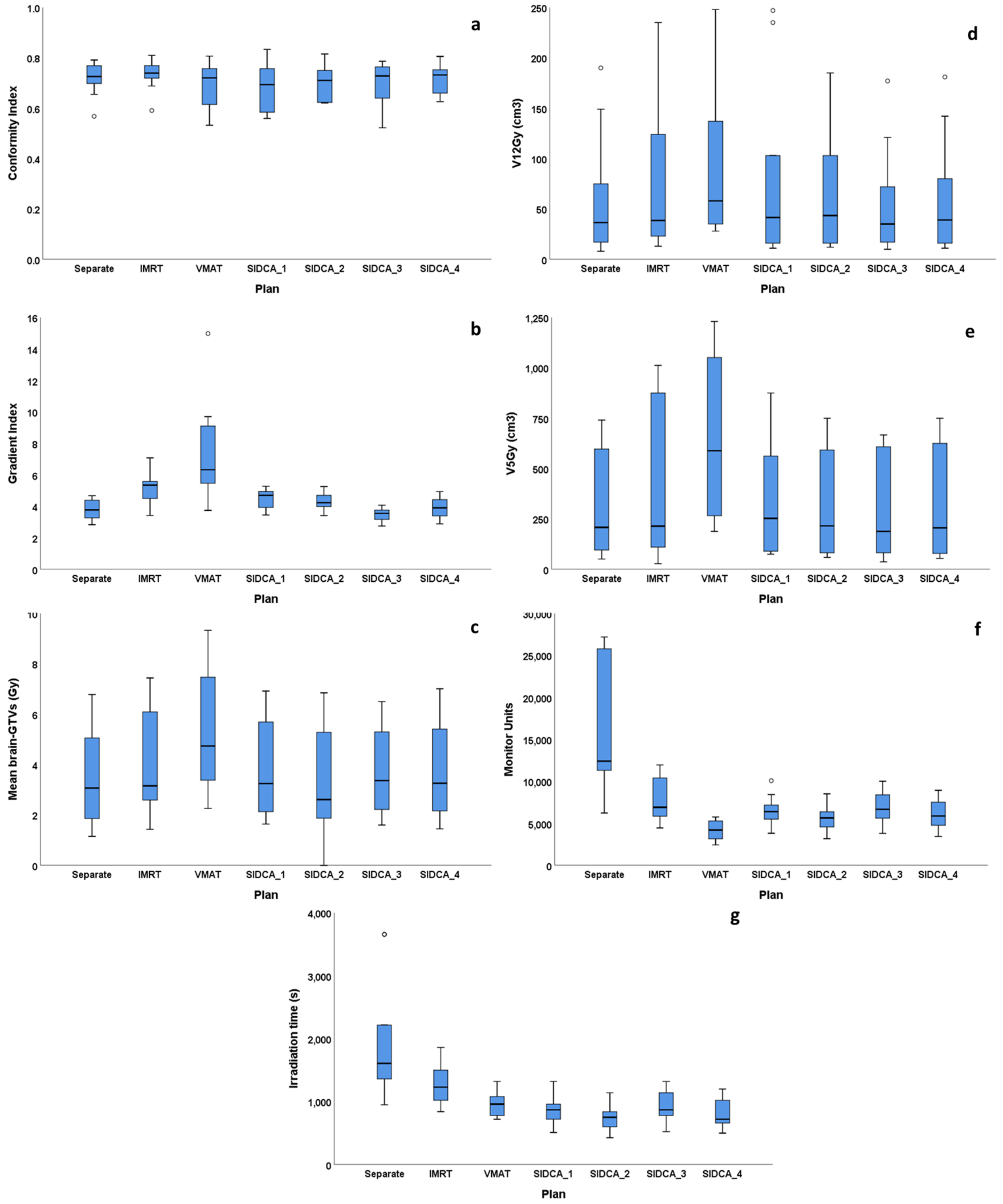

Figure S1. Statistical comparison of the crucial parameters for different planning techniques based on ten patients. Boxplots of the CI (**a**), the GI (**b**), the mean dose (**c**), the total V_{12Gy} (**d**) and V_{5Gy} (**e**) of the uninvolved brain, the number of MUs (**f**), and irradiation time (**g**) for separate-isocenter DCA (separate), IMRT, VMAT, MBM Elements v.1.6 with three couch angles (SIDCA_1), MBM Elements v.1.6 with five couch angles (SIDCA_2), MBM Elements v.2.0 with high NT (SIDCA_3), and low NT (SIDCA_4) plans for ten patients (# 9 and 12 excluded) irradiated in a single fraction. The central line of each box represents the median value, its upper and lower edges the 25th and 75th percentiles. The T-bars are called whiskers and extend to 1.5 times the height of the box values per planning technique. Outliers (circles) are defined as values that

do not fall in the whiskers. Extreme outliers (more than 3 box lengths from either hinge) are marked with an asterisk (*) on the boxplot.

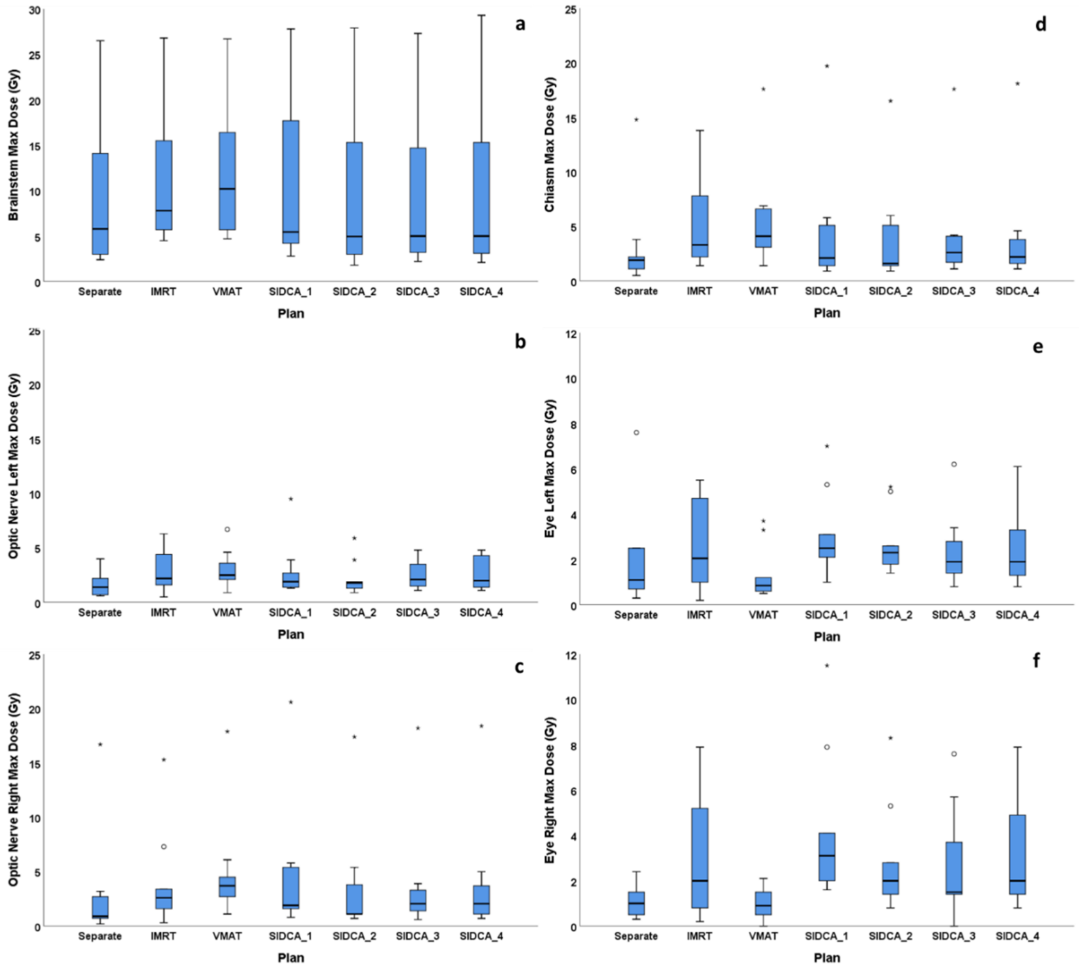

Figure S2. The boxplots of the maximum dose to brain stem (**a**), optic nerves (left (**b**), right (**c**)), chiasm (**d**), and eyes (left (**e**) right (**f**)) as a function of different plans for ten patients irradiated in a single fraction. Outliers (circles) are defined as values that do not fall in the whiskers. Extreme outliers are marked with an asterisk (*) on the boxplot.
